# Report from public health decision-maker interviews

**DOI:** 10.1093/eurpub/ckac129.629

**Published:** 2022-10-25

**Authors:** Keren Landsman

**Affiliations:** Ethik der Medizin, University of Augsburg, Augsburg, Germany

## Abstract

Report from NGO interviews on impact on vulnerable communities

Sylvia Agbih

Ethik der Medizin, University of Augsburg, Augsburg, Germany

Contact: sylvia.agbih@med.uni-augsburg.de

Report from school interviews

Quintus Sleumer

Ethik der Medizin, University of Augsburg, Augsburg, Germany

Contact: quintus.sleumer@uni-a.de

Report on selected key ethical issues arising from analysis

Verina Wild

Ethik der Medizin, University of Augsburg, Augsburg, Germany

Contact: verina.wild@uni-a.de

Building for the future: some thoughts on a comprehensive ethics assessment

Angus Dawson

Sydney Health Ethics, University of Sydney, Sydney, Australia

Contact: angus.dawson@sydney.edu.au

